# Incidence and Predictors of Unplanned Hospital Readmission after Percutaneous Coronary Intervention

**DOI:** 10.3390/jcm9103242

**Published:** 2020-10-10

**Authors:** Sinjini Biswas, Diem Dinh, Mark Lucas, Stephen J. Duffy, Angela L. Brennan, Danny Liew, Nicholas Cox, Voltaire Nadurata, Christopher M. Reid, Jeffrey Lefkovits, Dion Stub

**Affiliations:** 1 School of Public Health and Preventive Medicine, Monash University, Melbourne 3004, Australia; sinjini@gmail.com (S.B.); diem.dinh@monash.edu (D.D.); mark.lucas@monash.edu (M.L.); S.Duffy@alfred.org.au (S.J.D.); angela.brennan@monash.edu (A.L.B.); danny.liew@monash.edu (D.L); christopher.reid@curtin.edu.au (C.M.R.); jeffrey.lefkovits@mh.org.au (J.L.); 2 Department of Cardiology, The Alfred Hospital, Melbourne 3004, Australia; 3 Department of General Medicine, The Alfred Hospital, Melbourne 3004, Australia; 4 Department of Cardiology, Western Health, Melbourne 3021, Australia; nicholas.cox@wh.org.au; 5 Department of Medicine—Western Health, The University of Melbourne, Melbourne 3021, Australia; 6 Department of Cardiology, Bendigo Health, Bendigo 3550, Australia; v.nadurata@bendigohealth.org.au; 7 School of Public Health, Curtin University, Perth 6102, Australia; 8 Department of Cardiology, Royal Melbourne Hospital, Melbourne 3050, Australia; 9 Baker IDI Heart and Diabetes Institute, Melbourne 3004, Australia

**Keywords:** percutaneous coronary intervention, readmissions, outcomes

## Abstract

Unplanned readmissions to hospital after percutaneous coronary intervention (PCI) pose a significant burden to the healthcare system and are potentially preventable. In this study, we sought to determine the incidence of, and risk factors for, unplanned hospital readmissions within 30 days following PCI. We prospectively collected data on 28,488 patients undergoing PCI between 2013 and 2019, who were enrolled in the state-wide multi-centre Victorian Cardiac Outcomes Registry. Patients’ data were then linked to data from the Victorian Department of Health administrative database that records statewide hospital admissions. Disease diagnosis codes were used to identify cause of readmission. Patients who had an unplanned readmission were further divided into those who had a cardiac vs. non-cardiac cause for readmission. Overall, 3059 patients (10.7%) had an unplanned hospital readmission within 30 days of PCI, of which 1848 patients (60.4%) were readmitted for primarily cardiac diagnoses. Independent predictors of both 30-day unplanned cardiac and non-cardiac readmissions post-PCI were female sex, having ≥1 admission in the 12 months prior to PCI, acute coronary syndrome presentation, having any in-hospital complication and being discharged on an oral anticoagulant (all *p* < 0.05). A stepwise increase in readmission risk was observed with increasing number of admissions from 1 to ≥4 admissions in the 12 months prior to PCI. In conclusion, a substantial proportion of patients undergoing PCI have unexpected readmissions to hospital in the 30 days following PCI. Targeted strategies for patients with risk factors for readmission may be useful to reduce this significant burden to the healthcare system.

## 1. Introduction

Unplanned readmission to hospital is an important problem, which results in a significant burden to the healthcare system and adverse patient outcomes [1,2]. Readmission rates after percutaneous coronary intervention (PCI) vary significantly, with 30-day readmissions ranging from 4.7% to 15.6% reported in the literature [3]. Hospital readmission rates are increasingly being utilised as a quality metric for hospital performance and, in certain countries, tied to government funding or associated with financial penalty. For example, in the British National Health Service, hospitals do not receive additional payments if patients are readmitted within 30 days [4]. In addition, public reporting of hospital unplanned post-PCI readmission rates has been implemented in some health systems [5]. There is, therefore, substantial interest in understanding the factors that contribute to readmission after PCI which may help to prevent readmissions and improve patient outcomes. There are several clinical registries that track outcomes after PCI across the world [6]. However, clinical registries face resource constraints when performing post-discharge follow-up, which can potentially be overcome by linkage to administrative datasets containing routinely collected hospital data. Furthermore, data linkage to routinely collected administrative data mitigates the potential error from patient under-reporting of hospitalizations and consequent under-estimation of readmission rates encountered by some previous studies. Data linkage may, therefore, help to create a richer, more accurate data source from which factors associated with post-discharge events such as readmission can be ascertained. In the present study, we aimed to link data from a large multi-centre PCI registry to routine hospital datasets to determine the incidence of and factors associated with unplanned hospital readmissions in the first 30 days following PCI.

## 2. Methods

We undertook a retrospective cohort study of all patients undergoing PCI between 1 January 2013 and 31 December 2017 who were enrolled in the Victorian Cardiac Outcomes Registry (VCOR) PCI module, a multi-centre PCI registry which has previously been described in detail elsewhere [7]. Briefly, demographic, clinical, procedural and in-hospital outcome data are recorded on case-report forms using standardized definitions for all fields. All 13 public and 17 private hospitals that perform PCI in the state of Victoria, Australia participate and contribute data to the registry. The primary ethics approval was granted by the ethics committee at The Alfred Hospital (approval number 47/12), and the study was also approved by each participating hospital, including the use of opt-out consent.

Patients were divided into three groups: those who had one or more unplanned readmission within 30 days of PCI for primarily cardiac issues, those who those who had one or more unplanned readmission within 30 days of PCI for primarily non-cardiac issues, and those who were not readmitted. Patients with both types of readmission who had at least one cardiac readmission were classified in the cardiac readmissions group. These groups were then compared for baseline, presentation and procedural characteristics, as well as in-hospital care.

Data on unplanned readmissions within 30 days of PCI were obtained by deterministic linkage to the Victorian Admitted Episodes Dataset (VAED). In addition, VAED was used to calculate the number of admissions each patient incurred in the 12 months prior to the index PCI. The VAED comprises routinely collected demographic and administrative data on all admitted patients in Victorian public and private hospitals. Basic clinical data based on International Classification of Diseases, 10th revision, Australian modification (ICD-10-AM) diagnosis codes were also collected [8]. The censor date for linkage with the VAED in this study was 1 February 2018. Of the 32,852 patients who were enrolled in VCOR between the study dates and were alive at 30 days post-PCI, 194 patients were excluded from linkage due to insufficient case information at the time of linkage. Therefore, linkage was attempted in 32,658 patients, on whom successful matching was achieved for 28,488 (87.2%). ICD-10-AM diagnosis codes recorded in the VAED were used to determine the primary reason for readmission. An unplanned hospital readmission was defined as any overnight admission to hospital (excluding only emergency department stays) which was not for an elective purpose or procedure as per the ICD-10-AM code used for the primary reason for admission. For example, admissions for patients having elective staged PCI were not characterised as unplanned. Unplanned cardiac readmissions were defined as those where the primary reason for readmission based on ICD-10-AM codes, was either ischaemic heart disease, pericarditis, valvular heart disease, heart failure, arrhythmia, collapse requiring device implantation or presentation with cardiac signs/symptoms without a specific diagnosis. Readmissions with all other primary diagnoses were determined as non-cardiac. With regards to in-hospital complications, major bleeding was defined as bleeding requiring a transfusion and/or prolonging the hospital stay and/or causing a drop in haemoglobin >3.0 g/dL, while new renal impairment was defined as an absolute rise in serum creatinine ≥44.2 mmol/L or ≥25% up to five days after the index PCI.

Continuous variables were expressed as mean ± standard deviation and were compared using *t*-tests or Mann–Whitney U tests, as appropriate. Categorical data were expressed as numbers and percentages and were compared using Pearson’s Chi-square test. To determine the independent predictors of 30-day unplanned cardiac and non-cardiac readmissions after PCI, multivariable logistic regression analysis was performed. In total, 24 clinically relevant variables were considered. Those with a *p* value of <0.1 on univariate analysis that were not co-linear were entered into a stepwise backward-selection modelling process for multivariable assessment. Complete case analysis was performed for purposes of multivariable modelling (i.e., patients with missing values were excluded). The proportion of missing values was <1% for all variables.

All statistical analyses were performed using Stata 15.1 software (StataCorp LP, College Station, TX, USA). *p* values of <0.05 were considered to be statistically significant.

## 3. Results

**Figure 1:**
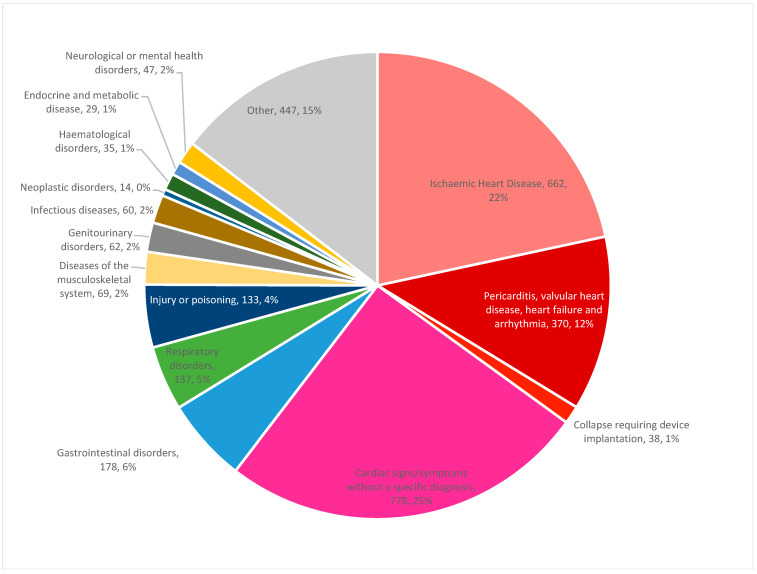
Causes of readmission.

**Figure 2:**
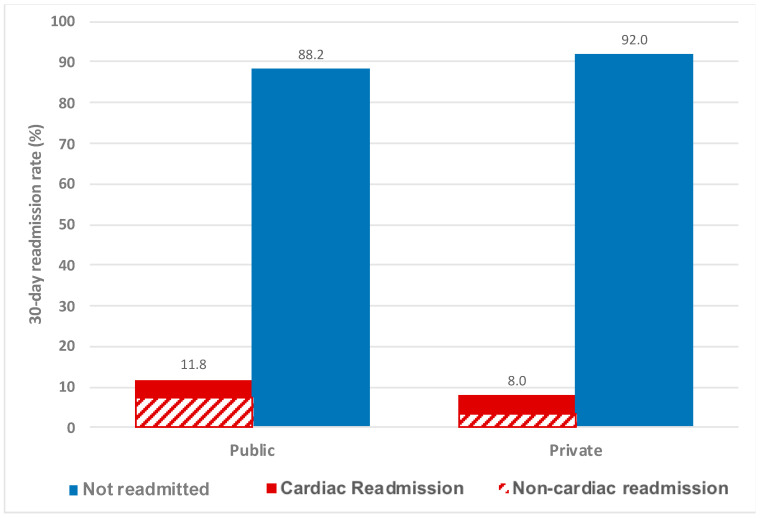
Comparison of readmission rates after PCI between public and private hospitals.

Our study cohort included 28,488 patients who underwent PCI during the study period and were successfully linked to VAED. Overall, the mean age was 67.2 ± 12.2 years and 6783 patients (23.8%) were female. Of the total cohort, 3059 patients (10.7%) had an unplanned hospital readmission. The primary cause of readmission was cardiac in nature in 1848 out of the 3059 patients (60.4%) who were readmitted. Of the cardiac readmissions, the vast majority were due to either ischaemic heart disease or cardiac signs and symptoms without a specific diagnosis. Out of the non-cardiac readmissions, the three most common causes for readmission were gastrointestinal disorders, respiratory disorders and injury or poisoning (Figure 1). Overall, the 30-day readmission rate was higher in public hospitals compared to private hospitals (11.8% vs. 8.0%; *p* < 0.001) (Figure 2).

Table 1 shows a comparison of the baseline characteristics of patients who had a cardiac or non-cardiac readmission to those who did not. Patients who had no readmissions were similar in age to those who had a cardiac readmission, while patients who had a non-cardiac readmission were older (mean age 66.2 ± 11.7 vs. 66.4 ± 12.0 vs. 71.1 ± 12.8 years; *p* < 0.001). Those who were readmitted were also more likely to be female than those who were not readmitted (23.0% vs. 30.5% vs. 31.0%; *p* < 0.001). There was a stepwise increase in the proportion of patients with comorbidities such as diabetes mellitus, stage 4–5 chronic kidney disease, peripheral vascular and cerebrovascular disease from the not readmitted to the cardiac readmission, and subsequently the non-cardiac readmissions group (all *p* < 0.001). Patients were more likely to be readmitted following PCI for acute coronary syndromes (ACS) than for stable angina. Of those who were readmitted, patients who had their index PCI for ACS were more likely to be readmitted for cardiac reasons, while those who had their PCI for stable angina were more likely to be readmitted for non-cardiac reasons. Patients who were readmitted were also more likely to have had an unsuccessful PCI, with cardiac readmissions being more frequent than non-cardiac ones (5.5% vs. 7.6% vs. 6.0%; *p* = 0.001).

Table 2 shows a comparison of discharge medications and in-hospital complications between the groups. Patients who were readmitted for either primarily cardiac or non-cardiac reasons, were more likely to have experienced in-hospital post-procedural complications such as new renal impairment, peri-procedural myocardial infarction and unplanned target vessel/lesion revascularisation, compared to those not readmitted (all *p* < 0.03). Patients who were readmitted were also more likely to have had a longer length of stay (≥3 days; *p* < 0.001) and be discharged on oral anticoagulants (6.8% vs. 9.3% vs. 13.1%; *p* < 0.001).

The results of multivariable logistic regression analysis to identify independent predictors of 30-day unplanned readmissions post PCI are shown in Table 3. Independent predictors of both 30-day cardiac and non-cardiac readmissions post-PCI were female sex, having ≥1 admission in the 12 months prior to PCI, presentation with ACS, having any in-hospital complication and being discharged on an oral anticoagulant (all *p* < 0.05). A stepwise increase in the odds ratio for both cardiac and non-cardiac readmissions was observed with each extra admission between 1 and ≥4 admissions in the 12 months prior to PCI (*p* < 0.001). Presentation with cardiogenic shock (OR 1.52, 95% CI 1.02–2.24); *p* = 0.04), having an unsuccessful PCI (OR 1.32, 05% CI 1.05–1.66; *p* = 0.02) and left ventricular systolic dysfunction (*p* < 0.03) were independent predictors of cardiac readmissions but not non-cardiac readmissions. Age >60 years (*p* < 0.001) and history of diabetes mellitus (*p* = 0.02) were found to be independent predictors of non-cardiac readmissions only.

## 4. Discussion

This large multi-centre study of patients undergoing PCI has shown that approximately 1 in 10 patients have an unplanned hospital readmission in the first 30 days following PCI. The majority of readmissions were cardiac in aetiology, with only 39.6% of readmissions being primarily for a non-cardiac problem. Having one or more admissions in the 12 months prior to PCI was consistently found to be an independent predictor of both cardiac and non-cardiac readmissions post PCI. A stepwise increase in the likelihood of readmission with an increasing number of admissions prior to PCI was also observed. Other independent predictors of 30-day readmission were female sex, having PCI for ACS, having any in-hospital complication and being discharged on an oral anticoagulant.

**Figure 3:**
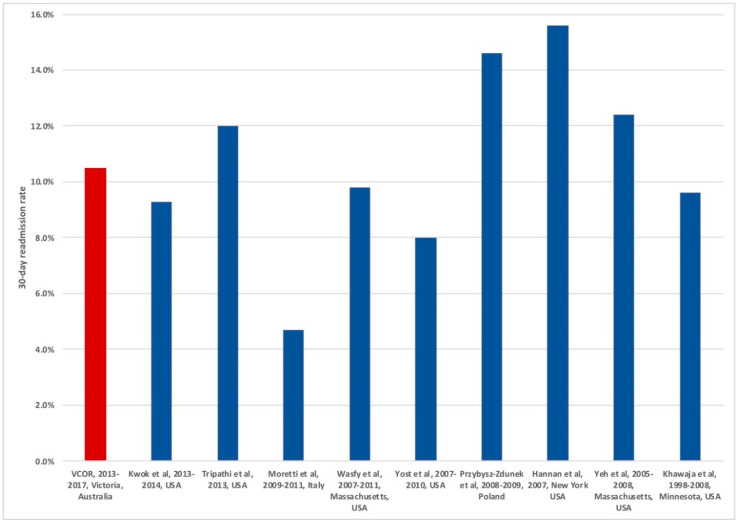
Comparison of 30-day readmission rates post-percutaneous coronary intervention across various studies. Note: Studies on x-axis denoted by first author, study inclusion dates and location of hospitals studies; the present study is highlighted in red.

Thirty-day readmission rates are increasingly being seen as a performance measure. Overseas, readmission rates are already being publicly reported in some countries and it has been suggested locally that a similar practice could be followed locally [5,9]. Therefore, there is keen interest from clinicians and hospital managers to identify and understand factors associated with 30-day readmission rates. Our 30-day readmission rates are within the range of readmission rates reported from cohorts overseas (Figure 3) [3,10,11,12]. Studies that have reported lower post-PCI 30-day readmission rates compared to ours have mostly had a smaller proportion of patients with ACS, which are known predictors of readmission [13,14,15]. Most of these studies were also each performed using data from 1 or 2 hospitals, while our study included 30 public and private hospitals, and is therefore more reflective of wider practice across Victoria. In addition, other studies included patients readmitted for planned staged PCI, whereas our study only included unplanned readmissions [14]. Of note, readmission rates were lower for patients having their index PCI at a private hospital, compared to a public hospital. Similar findings have been reported for readmissions for heart failure as well as after orthopaedic surgery [16,17]. Previous VCOR reports have suggested that patients undergoing PCI at private hospitals may be of lower acuity (higher proportion of PCI for stable angina and lower proportion of PCI for ACS compared to public hospitals), but further exploration is warranted [18].

We found that most readmissions were primarily due to cardiac diagnoses. This is similar to several previous studies, such as those by Curtis et al. and Hannan et al., which found that the majority of readmissions were due to primary cardiac diagnoses, even when staged PCI procedures were excluded [2,19]. Conversely, a more contemporaneous study by Kwok et al. found that non-cardiac causes for readmission were more common [20]. However, in that study, the authors included non-specific chest pain as a “non-cardiac” cause of readmission, unlike in our study, where any potential cardiac signs/symptoms without a specific diagnosis (including non-specific chest pain) were included as a cardiac readmission, which may explain some of the differences in our results. Notably, a quarter of readmissions in our study were due to non-specific cardiac symptoms or signs without a diagnosis. These readmissions may be potentially preventable by instituting nurse-led coordinated disease management programmes in post-PCI patients that include intensified patient education on recognising and managing angina, and early post-discharge follow-up. While such programmes have been shown to reduce the number of hospital admissions, and improve compliance, self-care behaviour and quality of life in patients with heart failure, their use is less widespread among patients undergoing PCI [21,22]. Implementation of such programmes may, therefore, help to reduce the burden of cardiac readmissions.

While most of the factors that we identified as being associated with 30-day readmission are not readily modifiable, they do highlight the subset of patients that are at high risk of readmission. Interestingly, age >60 years was found to be an independent predictor of non-cardiac but not cardiac readmissions. This may relate to older patients undergoing PCI having a greater burden of comorbidities, which have been shown to be more strongly associated with non-cardiac rather than cardiac readmissions [20]. Our study is, to our knowledge, the first to identify the number of admissions prior to PCI as a strong independent predictor of 30-day post-PCI readmissions. Several studies examining 30-day readmission after an admission for heart failure have shown that the number of recent admissions before the index stay was associated with an increased risk of readmission [23]. One of the largest studies to demonstrate this, by Hummel et al., showed a graded increase in the risk of readmission for patients who had one prior admission to two or more prior admissions, before the index stay for heart failure, similar to the findings of our study [24]. Similarly, a prior Australian study had demonstrated that patients with two or more emergency department presentations prior to hospitalisation with a stroke or transient ischaemic attack were more likely to be readmitted to hospital over the next year [25]. Identifying these high-risk patients is currently challenging, as information on the number of admissions a patient has previously had is not always readily available to clinicians in a timely manner. However, the introduction of large population-based electronic health records such as My Health Record in Australia may help clinicians to identify and provide targeted intervention to this group of high-risk patients, who may potentially benefit from more intensive post-discharge follow-up to prevent readmission [26]. Indeed, improved in-hospital education on cardiac symptoms and early follow-up for patients deemed to be high-risk for readmission following PCI have previously been shown to be effective in reducing 30-day readmission rates [27].

Our study has a number of limitations. Firstly, this was an observational study of patients enrolled in a PCI registry, and therefore we were unable to account for all possible factors associated with readmission following PCI such as frailty and comorbidities including cancer, dementia and depression. Secondly, readmissions outside the state of Victoria were not captured in our study, although we anticipate that this number would be small. Thirdly, classification of a readmission as planned or unplanned and cardiac or non-cardiac was dependent on coding quality by participating hospitals, which may not be uniform across all hospitals. Finally, practices that have a significant impact on readmission rates post-PCI, such as pre-discharge education on cardiac symptoms, may differ between health systems, and therefore our results may not be generalisable to other health systems internationally.

## 5. Conclusions

In conclusion, our study demonstrates that 30-day unplanned hospitals readmissions after PCI pose a significant burden to the health system. Most readmissions are primarily due to cardiac diagnoses. The most important predictive factor for both cardiac and non-cardiac readmissions appears to be the number of hospital admissions in the 12 months prior to PCI. This is valuable information for hospitals, as treatment and discharge policies, as well as outpatient follow-up timetables, may need to be tailored to patients at high risk for readmission following PCI.

## Figures and Tables

**Table 1:** Demographic and procedural characteristics by 30-day hospital readmission status.

	Not Readmitted (*n* = 25,423)	Any Cardiac Readmission (*n* = 1848)	All Non-Cardiac Readmissions (*n* = 1211)	*p*
**Baseline characteristics**				
Median age, years ± SD	66.2 ± 11.7	66.4 ± 12.0	71.1 ± 12.8	<0.001
Female	5844 (23.0)	564 (30.5)	375 (31.0)	<0.001
Body mass index groups:				
Underweight	165 (0.7)	15 (0.8)	14 (1.2)	<0.001
Normal	5422 (21.6)	457 (25.1)	326 (27.2)	
Overweight	10,342 (41.2)	701 (38.5)	439 (36.7)	
Obese	9144 (36.5)	649 (35.6)	418 (34.9)	
Aboriginal and/or Torres Strait Islander	104 (0.4)	10 (0.6)	7 (0.6)	0.50
Diabetes mellitus	5427 (21.3)	425 (23.0)	315 (26.0)	<0.001
Peripheral vascular disease	833 (3.3)	80 (4.3)	60 (5.0)	<0.001
Previous cerebrovascular disease	915 (3.6)	105 (5.7)	72 (5.9)	<0.001
Previous percutaneous coronary intervention	5557 (21.9)	349 (18.9)	219 (18.1)	<0.001
Previous coronary artery bypass graft surgery	1971 (7.8)	150 (8.1)	102 (8.4)	0.61
Stage 4–5 chronic kidney disease (estimated glomerular filtration rate (eGFR) < 30 mL/min/1.73 m^2^)	445 (1.9)	54 (3.1)	63 (5.6)	<0.001
Left ventricular ejection fraction (LVEF):				
LVEF ≥50	14,744 (67.1)	1035 (63.3)	591 (55.6)	<0.001
LVEF 45–49	4284 (19.5)	297 (18.2)	248 (23.4)	
LVEF 35–44	2099 (9.6)	208 (12.7)	143 (13.5)	
LVEF <35	843 (3.8)	94 (5.8)	80 (7.5)	
Number of admissions within the previous 12 months:				
0	20,196 (79.4)	1306 (70.7)	789 (65.2)	<0.001
1	3749 (14.7)	311 (16.8)	238 (19.7)	
2	953 (3.7)	110 (6.0)	88 (7.3)	
3–5	480 (1.9)	90 (4.9)	81 (6.7)	
>5	45 (0.2)	31 (1.7)	15 (1.2)	
**Presentation characteristics:**				
Stable angina	11,499 (45.2)	470 (25.4)	396 (32.7)	<0.001
Non-ST elevation acute coronary syndrome	8512 (33.5)	853 (46.2)	486 (40.1)	
ST-elevation myocardial infarction	5412 (21.3)	525 (28.4)	329 (27.2)	
Cardiogenic shock	329 (1.3)	37 (2.0)	39 (3.2)	<0.001
Intubated-out-of-hospital cardiac arrest	41 (0.2)	3 (0.2)	5 (0.4)	0.12
Out-of-hours presentation	12,226 (48.1)	952 (51.5)	607 (50.1)	0.008
**Procedural characteristics:**				
Radial/brachial access	13,038 (51.3)	927 (50.2)	577 (47.6)	0.03
Femoral access	12,385 (48.7)	921 (49.8)	634 (52.4)	
Multi-vessel disease	1689 (6.6)	117 (6.3)	84 (6.9)	0.80
ACC/AHA B2/C lesion	14,252 (56.1)	1047 (56.7)	727 (60.0)	0.02
Chronic total occlusion lesion treated	966 (3.8)	62 (3.4)	46 (3.8)	0.62
Unprotected left main vessel lesion	257 (1.0)	19 (1.0)	12 (1.0)	0.99
Drug-eluting stent used	20,894 (82.2)	1421 (76.9)	940 (77.6)	<0.001
Glycoprotein IIb/IIIa inhibitor used	2756 (10.8)	246 (13.3)	161 (13.3)	<0.001
Unsuccessful percutaneous coronary intervention	1407 (5.5)	140 (7.6)	73 (6.0)	0.001

**Table 2:** In-hospital stay and complications and discharge medications by 30-day hospital readmission status.

	Not Readmitted (*n* = 25,423)	Any Cardiac Readmission (*n* = 1848)	All Non-Cardiac Readmission (*n* = 1211)	*p*
Median length of stay, days [IQR]	2 (1–4)	3 (2–5)	4 (2–6)	<0.001
Length of stay <3 days	13,183 (51.9)	661 (35.8)	394 (32.5)	<0.001
Length of stay ≥3	12,240 (48.1)	1187 (64.2)	817 (67.5)	
**Discharge destination:**				
Home	24,382 (95.9)	1724 (93.3)	1106 (91.3)	<0.001
Hospital in the home	109 (0.4)	7 (0.4)	7 (0.6)	
Rehab	445 (1.8)	40 (2.2)	54 (4.5)	
Local or referring hospital	380 (1.5)	57 (3.1)	32 (2.6)	
Tertiary referral centre	107 (0.4)	20 (1.1)	12 (1.0)	
**In-hospital complications**				
New renal impairment	426 (2.3)	58 (3.8)	64 (6.5)	<0.001
Cardiogenic shock	164 (0.6)	32 (1.7)	19 (1.6)	<0.001
Peri-procedural myocardial infarction	142 (0.6)	25 (1.4)	16 (1.3)	<0.001
Unplanned PCI for target vessel revascularisation	53 (0.2)	9 (0.5)	5 (0.4)	0.03
Target lesion revascularisation	106 (0.4)	17 (1.0)	9 (0.8)	0.003
Unplanned coronary artery bypass surgery	101 (0.4)	11 (0.6)	14 (1.2)	<0.001
Stent thrombosis	38 (0.1)	2 (0.1)	3 (0.2)	0.61
Major bleeding	156 (0.6)	14 (0.8)	20 (1.7)	<0.001
Stroke	49 (0.2)	5 (0.3)	3 (0.2)	0.72
Any of the above in-hospital complications	621 (2.5)	90 (5.0)	69 (5.9)	<0.001
**Discharge medications**				
Aspirin	24,727 (97.5)	1791 (97.3)	1165 (96.4)	0.09
Thienopyridine	13,802 (54.5)	900 (49.0)	625 (51.8)	<0.001
Ticagrelor	11,048 (43.6)	889 (48.5)	548 (45.5)	<0.001
Beta-blocker	17,666 (70.1)	1382 (75.5)	898 (74.6)	<0.001
Angiotensin converting enzyme inhibitors/angiotensin receptor blockers	18,233 (72.3)	1325 (72.4)	880 (73.2)	0.82
Any lipid lowering therapies	23,791 (94.2)	1731 (94.2)	1119 (92.9)	0.21
Oral anticoagulants	1728 (6.8)	170 (9.3)	158 (13.1)	<0.001

**Table 3:** Independent predictors of 30-day cardiac and non-cardiac readmissions post-PCI.

		Any Cardiac Readmission		All Non-Cardiac Readmissions	
		Odds Ratio (95% CI)	*p*	Odds Ratio (95% CI)	*p*
Age group					
	51–60 years	1 (ref.)		1 (ref.)	
	<40 years	0.78 (0.53–1.16)	0.22	0.64 (0.34–1.20)	0.16
	41–50 years	1.18 (0.98–1.42)	0.08	1.03 (0.77–1.39)	0.83
	61–70 years	0.94 (0.81–1.09)	0.38	1.45 (1.18–1.78)	<0.001
	71–80 years	0.97 (0.83–1.14)	0.72	2.00 (1.63–2.45)	<0.001
	>80 years	1.15 (0.94–1.40)	0.18	2.47 (1.94–3.13)	<0.001
Sex					
	Male	1 (ref.)		1 (ref.)	
	Female	1.46 (1.30–1.64)	<0.001	1.29 (1.12–1.49)	<0.001
Diabetes mellitus		1.08 (0.95–1.23)	0.25	1.21 (1.04–1.40)	0.02
Peripheral vascular disease		1.21 (0.92–1.58)	0.18	1.03 (0.76–1.42)	0.83
Previous stroke		1.37 (1.08–1.74)	0.009	1.18 (0.89–1.56)	0.25
Previous PCI		0.87 (0.75–1.01)	0.06	0.76 (0.63–0.90)	0.002
Previous CABG		1.02 (0.82–1.27)	0.88	0.91 (0.71–1.18)	0.48
Presentation diagnosis					
	Stable angina	1 (ref.)		1 (ref.)	
	NSTEACS	2.62 (2.23–3.09)	<0.001	1.71 (1.42–2.06)	<0.001
	STEMI	2.79 (2.29–3.38)	<0.001	1.76 (1.40–2.22)	<0.001
Cardiogenic shock at presentation		1.52 (1.02–2.24)	0.04	1.19 (0.96–1.47)	0.11
Number of admissions within previous 12 months:					
	0	1 (ref.)		1 (ref.)	
	1	1.50 (1.29–1.73)	<0.001	1.71 (1.44–2.02)	<0.001
	2	1.86 (1.47–2.36)	<0.001	2.20 (1.69–2.85)	<0.001
	3	3.36 (2.56–4.40)	<0.001	4.58 (3.47–6.06)	<0.001
	≥4	11.83 (6.89–20.31)	<0.001	8.34 (4.18–16.66)	<0.001
Drug-eluting stent used		0.88 (0.77–1.01)	0.08	0.87 (0.74–1.03)	0.11
Unsuccessful PCI		1.32 (1.05–1.66)	0.02	1.05 (0.86–1.29)	0.62
Any in-hospital complication		1.72 (1.32–2.23)	<0.001	1.71 (1.26–2.31)	0.001
Left ventricular ejection fraction (LVEF):					
	LVEF ≥50%	1 (ref.)		1 (ref.)	
	LVEF 45–49%	1.22 (1.04–1.45)	0.02	1.07 (0.90–1.27)	0.44
	LVEF 35–44%	1.33 (1.09 1.64)	0.006	1.14 (0.89–1.45)	0.30
	LVEF <35%	1.39 (1.06–1.82)	0.02	1.19 (0.96–1.47)	0.11
Discharged on oral anticoagulant		1.26 (1.05–1.53)	0.02	1.50 (1.22–1.84)	<0.001

PCI = percutaneous coronary intervention; CABG = coronary artery bypass graft surgery; NSTEACS = Non-ST elevation acute coronary syndromes; STEMI = ST-elevation myocardial infarction.
